# Role of ABCA7 in Human Health and in Alzheimer’s Disease

**DOI:** 10.3390/ijms22094603

**Published:** 2021-04-27

**Authors:** Shiraz Dib, Jens Pahnke, Fabien Gosselet

**Affiliations:** 1 UR2465, LBHE-Blood–Brain Barrier Laboratory, University Artois, 62300 Lens, France; shiraz_deeb@hotmail.com; 2 Department of Neuro-/Pathology, University of Oslo and Oslo University Hospital, Sognsvannsveien 20, 0372 Oslo, Norway; jens.pahnke@medisin.uio.no; 3 LIED, University of Lübeck, Ratzenburger Allee 160, 23538 Lübeck, Germany; 4 Department of Pharmacology, Faculty of Medicine, University of Latvia, Jelgavas iela 3, 1004 Riga, Latvia; 5 Department of Bioorganic Chemistry, Leibniz-Institute of Plant Biochemistry, Weinberg 3, 06120 Halle, Germany

**Keywords:** ABCA7, Alzheimer’s disease, phagocytosis, cholesterol, Aβ peptides

## Abstract

Several studies, including genome wide association studies (GWAS), have strongly suggested a central role for the ATP-binding cassette transporter subfamily A member 7 (ABCA7) in Alzheimer’s disease (AD). This ABC transporter is now considered as an important genetic determinant for late onset Alzheimer disease (LOAD) by regulating several molecular processes such as cholesterol metabolism and amyloid processing and clearance. In this review we shed light on these new functions and their cross-talk, explaining its implication in brain functioning, and therefore in AD onset and development.

## 1. Introduction

ATP-binding cassette (ABC) transporters are present in all living organisms. In humans, this family comprises 49 members that are classified based on their sequence homologies and their conserved nucleotide binding domains (NBDs) into seven subfamilies, designated from A to G. These transporters play a significant role by transporting ions, small organic or inorganic molecules, peptides, proteins and lipids from a cellular compartment to another one or across the cell membranes [1,2]. Importantly, their physiological functions strongly depend on their cellular localization (membrane, endoplasmic reticulum, Golgi, etc.) as well as the organ and cell type in which they are expressed. For example, members of the ABCB and ABCC subfamilies are expressed in physiological barriers such as intestine and blood–brain interfaces to restrict the entry or mediate the exit of harmful endogenous molecules or xenobiotics [3,4]. Overexpression of these ABC transporters has been reported in several cancers and is responsible for the multidrug resistance (MDR) phenotype of cancer cells [5]. Members of the ABCG and A subfamilies mediate cholesterol and lipid efflux from cells to low- or high-density lipoproteins and are highly expressed by cells involved in lipid homeostasis such as macrophages or hepatocytes.

It is therefore not surprising that ABC transporter deficiencies lead to major diseases including but not limited to atherosclerosis, cystic fibrosis or neurodegenerative diseases such as Alzheimer’s disease (AD) [2,6,7]. AD is the most common form of dementia in the world. This disease is mainly characterized by brain atrophy as a consequence of the deposition of amyloid-β (Aβ) peptides and tau protein hyperphosphorylation and aggregation [8]. Since the early 2000s, several ABC transporters such as ABCB1, ABCA1, ABCG2, ABCC1 and ABCG4 are under scrutiny in AD because of their in vitro and in vivo involvement in Aβ peptide synthesis and/or its deposition. These different contributions in the AD field are already summarized elsewhere [4,7,9,10].

**Figure 1:**
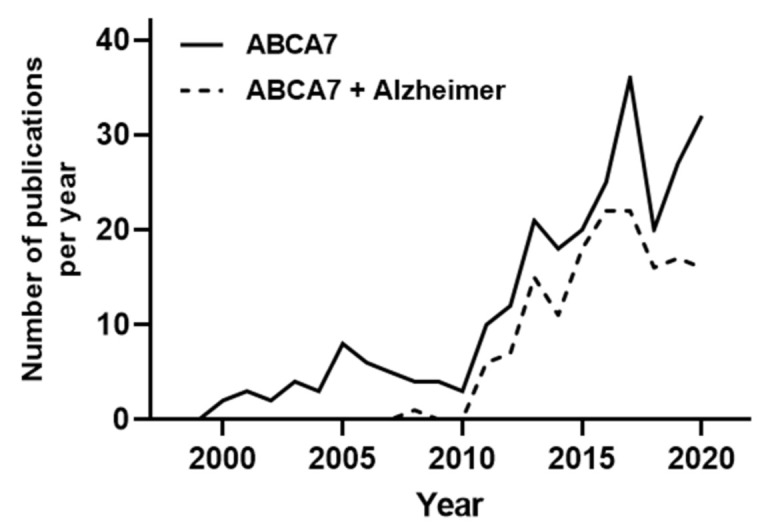
Number of searchable publications in Pubmed per year by the terms specified in the legend. For the “ABCA7 + Alzheimer” search, a second analysis of the obtained publications was performed to exclusively select studies and review investigating the role of ABCA7 in AD. Data source: Pubmed. Search date 31/12/2020. Y-axis shows number of publications, X-axis shows years

The ATP-Binding cassette transporter subfamily A member 7 (ABCA7) was first identified in the early 2000s [11]. As shown in Figure 1, ABCA7 was poorly studied until 2011. These studies mainly investigated ABCA7 involvement in peripheral cholesterol metabolism and phagocytosis processes. In 2011, Hollingworth et al. highlighted a very strong association between ABCA7 polymorphisms and AD patients in a genome-wide association study (GWAS) [12].

Since then, the number of projects studying ABCA7 have been considerably increased, and almost all of them have been realized with the objective to decipher the molecular role of ABCA7 in AD (Figure 1). 

This review aims to summarize the first roles of ABCA7 identified in the periphery, but also to describe the most recent findings demonstrating its significant contribution in brain functioning and in AD onset and development.

## 2. ABCA7 Expression Pattern and Structure

### 2.1. Tissue Localization of ABCA7

ABCA7 was first cloned in 2000 by Kaminski and colleagues from human macrophages [11]. Distribution/expression of the ABCA7 mRNA in human tissues demonstrated that it is highly expressed in myelo-lymphatic tissues (peripheral leucocytes, thymus, spleen, bone marrow, fetal tissues) [11]. Preferential and high expression of *Abca7* mRNA in lymphomyeloid tissues was immediately confirmed in mice and rats [13,14], strongly suggesting a key role of this transporter in hematopoietic cell lineages. More recently, analysis based upon ImmGen Deep RNA-seq data showed that *Abca7* is one of the most highly expressed ABC transporter in a purified population of mouse immune cells like follicular B cells, NK cells, peritoneal macrophages, thus highlighting a role for ABCA7 in immunity [15]. In addition, it appears that ABCA7 expression is dependent of the differentiation state of the cells since higher expressions were measured in differentiated macrophages comparing to monocytes [11].

Interestingly, no ABCA7 mRNA signal was initially detected in total human brain samples but this result was later refuted by several groups demonstrating mRNA and protein expression of ABCA7 in human and murine samples of the different cell types present in the brain [16,17,18,19,20,21,22,23]. Therefore, it is now largely demonstrated that ABCA7 is expressed in neurons, astrocytes, microglia, endothelial cells of the blood–brain barrier (BBB), brain pericytes, not only in mice but also in humans ([16,17,18,19,20,21,22,23,24]—http://www.brainrnaseq.org/, http://www.celltypes.org/ and https://www.proteinatlas.org/ENSG00000064687-ABCA7/brain, accessed on 31 December 2020).

### 2.2. ABCA7 Alternative Splicing

There are two isoforms of ABCA7, arising as a result of alternative splicing, both of which are detected in the human brain [21,25]. The splicing variant is named Type II ABCA7 and gives a protein with 28 amino acids in the N terminal tail instead of the 166 amino acids present in the full-length ABCA7. These variants show a tissue-dependent expression pattern and differential cellular sub-localization. While full length ABCA7 has been detected on the cell surface and intracellularly, the so-called Type II ABCA7 splice variant is only detected in the endoplasmic reticulum [21]. Northern blot analysis performed with human tissue samples showed that Type II ABCA7 is abundant in lymph node, spleen, thymus and trachea whereas full ABCA7 is strongly expressed in brain and bone marrow [21]. Thus, it is possible that these variant proteins have different biological functions.

### 2.3. ABCA7 Structure

**Figure 2:**
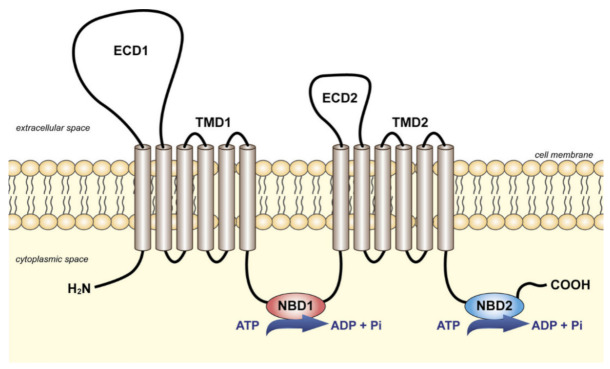
Schematic representation of the full ABC transporters of the subfamily A. Two transmembrane domains (TMDs) are connected with two cytoplasmic nucleotide-binding domains (NBDs). In addition, members of subfamily A are characterized by two large extracellular domains (ECD1 and ECD2). Reprinted from [26] with permission from Elsevier.

Functional ABC transporters are commonly composed of four domains—two transmembrane domains (TMDs) connected with two cytoplasmic nucleotide-binding domains (NBDs). TMDs are variable in sequence and are responsible for substrate interaction and translocation. NBDs are highly conserved and have characteristic motifs such as Walker A and Walker B, allowing to bind and hydrolyze ATP. As shown in Figure 2, full ABC transporters like transporters of subfamilies A and C have 2 TMDs. ABC transporters of the D and G subfamilies such as ABCG1 or ABCG2 display only one TMD and one NBD. They are therefore considered as half-transporters and have to homo- or hetero-dimerize to be functional. ABC transporters are classified based on their NBD domain but it was recently suggested that it would be more pertinent to use a TMD-based classification system [1].

ABCA7 is predicted to be a full ABC transporter, as presented in Figure 2. The transport mechanism of these transporters is controlled by alternating the conformation of TMD, through which the transporter switches between inward- and outward-facing states. Recently, alternative mechanisms have been proposed or identified, suggesting that this process remains under debate [27]. In all cases, the exact transport mechanism of ABCA7 is uncharacterized, and, despite the fact that single-particle cryoelectron microscopy (cryo-EM) structure of ABCA1 has been recently obtained [28], the structure of ABCA7 is still unknown.

Sequence comparisons made with other ABC transporters showed high ABCA7 homology with ABCA1 (54%), ABCA2 (45%), ABCA3 (41%) and ABCA4 (49%) [11]. Because these latter are involved in lipid transport, it was rapidly assumed that ABCA7 was also able to transfer lipid from cell membranes to lipoproteins.

## 3. ABCA7 and the Lipid Metabolism

### 3.1. Role of ABCA7 in Lipid Release and Trafficking

As indicated above, ABCA7 shares high amino sequence homology with other ABC transporters mediating lipid release from cell membranes, and in particular with ABCA1. This latter is the most studied ABC transporter in this cellular process and its role in cholesterol efflux to high-density lipoproteins (HDL) is very well characterized [29,30,31]. Mutations in the *ABCA1* gene cause an inherited disease named Tangier disease in which the cholesterol transfer from cells to HDL is decreased, leading to lower ApoA-I synthesis as well as low levels of plasmatic HDL [32]. Consequently, this lipid homeostasis imbalance promotes lipid accumulation inside vascular cells and macrophages, thus provoking coronary artery disease and atherosclerosis in patients [27,33,34].

On this basis, *Abca7*^−/−^ mice were generated to further characterize the role of this ABC transporter in cholesterol metabolism. Interestingly, only transgenic female mice showed slight abnormalities in their cholesterol metabolism when compared with the wild-type littermates [16]. Indeed, female *Abca7*^−/−^ mice showed weight gain identical to female controls but less white adipose tissue, plasmatic HDL and cholesterol [16]. No significant differences have been measured in males. In the same study, no alteration of the cholesterol or phospholipid efflux was measured in macrophages purified from the *Abca7*^−/−^ mice. However, further in vitro studies using different transfected cell types reported that ABCA1, ABCA7 binds ApoA-I, and ApoE, to transfer cholesterol and to generate HDL [18,19,20]. Of note, this efflux is marginal when compared with cholesterol efflux mediated by ABCA1. On the contrary, ABCA7 is more prone to transfer phospholipids to HDL, in particular sphingomyelin, and (lyso)phosphatidylcholine [18,19,35]. Importantly, almost all of these studies were done by artificially upregulating or downregulating *Abca7* expression that, in turn, provokes a modification of ABCA1 expression, probably by a compensatory mechanism [20,36]. This effect is also reciprocal because the decrease of *Abca1* expression increases *Abca7* level [37]. Abe-Dohmae et al. demonstrated that this compensatory mechanism allows the transfer of cholesterol to lipoproteins when the *Abca1* gene is deficient or absent [35]. However, it remains still unknown why ABCA7 cannot compensate this deficiency in a Tangier disease patient. In light of all these data, it is now widely accepted that ABCA7 is specialized in phospholipid transfer to HDL whereas ABCA1 releases cholesterol.

It is also clear that *Abca7* transcriptional expression is closely regulated by cholesterol metabolism. BALB/3T3 cells loaded with cholesterol showed a significant downregulation of *Abca7* whereas an upregulation of *Abca1* was observed [37]. On the contrary, cholesterol-depleted cells displayed increased *Abca7* expression. In fact, ABCA7 expression is regulated by the sterol-responsive/regulatory element binding protein (SREBP), able to bind specific sterol regulatory element DNA sequences when mammalian cells lack cholesterol, thus triggering the expression of the mRNA of genes involved in cholesterol synthesis or lipoprotein uptake such as HMG-CoA reductase or LDLR, respectively [37]. Blocking HMG-CoA reductase activity with statins, thus blocking cholesterol synthesis, activates SREBP nucleus translocation and increases ABCA7 expression in murine macrophages [38]. Interestingly, *ABCA1* expression is controlled by the Liver X receptor (LXR) nuclear receptors [39], also acting as cholesterol sensors in mammalian cells, thus probably explaining why ABCA1 and ABCA7 expressions are regulated differently to modulate the intracellular pool of cholesterol.

ABCA7 is also implicated in lipid trafficking during keratinocyte differentiation and is reported as a ceramide homeostasis regulator. ABCA7 upregulation was detected in normal human epidermal keratinocytes and HaCaT cells undergoing in vitro differentiation in parallel with an increase in intracellular ceramide levels. In accordance with these results, ABCA7 overexpression in Hela cells showed a ceramide de novo synthesis activation with an increase in intracellular and cell surface ceramide expression [40].

Altogether, these data reinforced the hypothesis that ABCA1 and ABCA7 are closely linked to regulate the cellular lipid metabolism but the role of ABCA7 seems to be rather indirect or negligible when directly compared to ABCA1.

### 3.2. Role of ABCA7 in Phagocytosis and Immune Response

In their initial investigation about ABCA7 sequence genomic organization, Kaminski et al. also observed that ABCA7 is arranged in a head-to-tail array with the human minor histocompatibility antigen HA-1 in a common locus on the 19p13.3 chromosome [11]. Since HA-1 is implicated in host defense, and based on its physical proximity to ABCA7, the question about possible common regulatory and functional mechanisms between these two genes was quickly raised [11]. Another clue suggesting a role of ABCA7 in immunity is the high sequence homology of the ABCA7 gene with cell death proteins (*Ced*) genes. In *Caenorhabditis elegans*, *ced* genes are key players of engulfment of dying cells. Among all these proteins, CED-7 is required for CED-1 functioning. Interestingly, mammalian orthologues have been proposed for all *Ced* genes, based on their sequence homologies. ABCA1 and ABCA7 have been proposed as being orthologues for CED7 [41], and low-density lipoprotein receptor–related protein 1 (LRP1) has been suggested as a protein with similar function to CED-1 [42]. In macrophages, ABCA7 and LRP1 were relocalized together to the plasma membrane in the presence of apoptotic cells, thus promoting their engulfment. Macrophages blocked with an antibody against LRP1 or *Abca7*^+/-^ macrophages show both lower phagocytosis of these dying cells, as well as a decrease of the ERK phosphorylation, highlighting links between this ABC transporter, LRP1 and this signaling pathway [41]. ABCA7 expression and mediated phagocytosis are also increased when cholesterol synthesis is inhibited by statins that block HMG-CoA activity [38], demonstrating again strong relationships between cellular cholesterol pool and ABCA7 activity and expression.

As indicated above, ABCA7 is also able to interact with ApoA-I and HDL. Therefore, a potential contribution of (apo)lipoproteins in phagocytosis was investigated by Tanaka et al. They reported that extracellular HDL increases ABCA7-associated phagocytosis by stabilizing ABCA7, thus suggesting an involvement of the HDL components in the host defense system that deserves further investigation [38].

Therefore, a possible role of ABCA7 in host defense was clearly established and investigated by several studies reporting the ABCA7 implication in phagocytosis-mediated processes by macrophages instead of cholesterol regulation.

However, until 2011, no study clearly demonstrated a key role of ABCA7 in a human disease. When Hollingworth et al. published that single nucleotide polymorphisms (SNPs) in ABCA7 sequences were strongly associated with Alzheimer’s disease (AD) onset and development [12], a new field of investigation has emerged with the objectives to identify the exact function of ABCA7 in the brain as well as in the apparition and evolution of AD.

## 4. Roles of ABCA7 in Brain Functioning and in Alzheimer’s Disease (AD)

### 4.1. AD Pathology

AD is a neurodegenerative disease and the number one leading cause of dementia in the elderly [43]. Most AD patients show a sporadic form of the disease (late-onset AD, LOAD) whereas almost 1% of them show a familial early-onset form (FAD), with Mendelian inheritance (<60 years). Nevertheless, these two forms of AD are both characterized by accumulation of amyloid-β (Aβ) peptides and hyperphosphorylated tau protein, both provoking neuronal loss mainly in the hippocampus and cortex. Major AD symptoms are thus memory loss and behavioral complications [8].

Links between Aβ peptides and tau are still elusive despite a large number of studies, but it is now widely accepted that Aβ peptide deposition precedes the tau hyperphosphorylation by several decades [44]. In addition, FAD patients possess mutations in genes involved in Aβ peptide production, as, for example, in amyloid beta precursor protein (APP) sequence and in the enzyme responsible for APP cleavage. For this reason, it is compulsory to better understand the early molecular and cellular events controlling Aβ peptide synthesis and clearance, thus leading to the early step of the cerebral Aβ peptide accumulation.

All cells in the body are able to synthesize these peptides from APP but neurons remain the major sources of Aβ peptides [7]. APP is cleaved by at least three types of proteases (α-, β- and γ-secretases). To date, two processing pathways have been described—the amyloidogenic pathway generates Aβ peptides via β-secretase, whereas the anti-amyloidogenic pathway is initiated by α-secretase and prevents Aβ peptide generation. Interestingly, cellular cholesterol content can strongly influence these pathways and modulate Aβ synthesis, as discussed below and reviewed elsewhere [45].

Cerebral pools of Aβ peptides are cleared by several mechanisms, including phagocytosis processes occurring at the microglial cell level, and elimination across the blood–brain barrier (BBB). BBB physiology and functioning are summarized elsewhere and exhaustive reviews can be consulted for more details [7,10,46]. In brief, this physical and metabolic barrier is composed of the endothelial cells lining the brain microvessels. These cells are sealed together by tight junctions (TJs) in relation with adherent (AJs) and gap (GJ) junctions. Importantly, BBB endothelial cells also express a large panel of ABC transporters involved in the cerebral efflux of Aβ peptides but also restricting their entry to the CNS. Thus, a large body of evidence has confirmed in humans and in several animal models the involvement of P-gp (ABCB1), BCRP (ABCG2), MRP1 (ABCC1) and more recently ABCG4 (reviewed in [4,7]).

Noteworthy, some studies provide strong evidence that a decrease of amyloid clearance across the BBB or a decrease of phagocytosis by microglial cells might be involved in AD rather than an Aβ peptide overproduction by cleavage of the APP [47]. When analyzed in mouse models of AD and in patients, expression of ABCB1, ABCC1 and ABCG2 at the BBB are decreased. Restoration of these expressions increases amyloid clearance across the BBB, but also decreases the entry of peripheral Aβ peptides into the CNS, and thus reduces the brain Aβ burden and alleviates cognitive dysfunction [7,48,49,50,51]. However, despite these promising results, there is currently no cure for AD.

Because AD is also closely linked to the cholesterol metabolism and because the brain is one of the most cholesterol-rich organs of the body, researchers also focused their attention on ABCA1. In transgenic mice models, altering the cerebral cholesterol homeostasis by overexpressing or downregulating *Abca1* expression decreases or increases Aβ peptide synthesis and deposition, respectively [52,53,54].

It has also been largely demonstrated that environmental factors such as diet or lack of activity can promote AD onset. For example, animals fed with cholesterol-rich diets show increased production of Aβ peptides, whereas the use of statins decrease, in vivo and in vitro, this synthesis and deposition processes [45,55]. In addition, genetic factors are also largely involved as suggested since the early 2010s with the discovery of several gene polymorphisms in AD by GWAS. These studies confirmed that *ApoE4*, identified in the early 1990s, and is an important component of the low-density lipoproteins (LDL), remains the most strongly associated allele to AD. However, interestingly, almost 20 other genes have been identified, among them ABCA7, for which very little information in relation to its physiological and cellular functions has been identified so far.

Since 2011, genetic polymorphisms of *Abca7* have been reported in several dozens of genetic studies using samples coming from AD patients worldwide (reviewed in [56,57]). Further investigations of several of these variants demonstrated that they are responsible for an alteration of ABCA7 expression in AD brains. While a protective ABCA7 allele was identified, deleterious alleles are responsible for protein loss-of-function or ABCA7 downregulation or upregulation [56,57,58]. These ABCA7 loss-of-function mutations increase risk of AD by 80% in African ancestry populations and, risk of early AD onset by 100% to 400% in European ancestry populations [59,60,61]. Table 1 summarizes the AD-associated ABCA7 epigenetic and genetic variations, but also the available data obtained in patients regarding the amyloid and tau pathology as well brain morphology and clinical symptoms.

### 4.2. ABCA7 in Brain Functions

*Abca7* is mainly expressed by neurons and microglia in human and mouse brains [23,80,81,82]. Both isoforms described in Section 2.2 are observed by western blots in brain samples of healthy donors and AD patients [25]. Consequences of *Abca7* depletion on brain cholesterol homeostasis were first investigated in mice. Lipidomic analysis of forebrain samples from five male *Abca7*^−/−^ mice showed alterations in lipid content; of the 275 studied lipids, only 24 were significantly affected by the absence of expression of *Abca7* [83]. Among them, 12 subspecies in ethanolamine, three in phosphoglycerol, one in lysophosphatidylcholine, and two in shingomyelin were lower in brains of *Abca7*^−/−^ mice. Three subspecies in phosphatidylcholine, one in ceramide, three in sulfatide, and one in cerebroside were increased, thus suggesting that *Abca7* deficiency may significantly affect cerebral lipid metabolism.

The same study reported deficiencies in spatial memory in *Abca7*^−/−^ 20- to 22-month-old male and female mice [83] but these deficiencies were only observed in 19- to 20-week-old females in another study performed by Logge et al. [84]. In the same study, males and females displayed an altered novel object recognition but this was more pronounced in females [84]. No difference between genders or with wild type mice was measured when a battery of tests for behavior was realized. Indeed, *Abca7*^−/−^ mice showed the same sensory abilities, neurological reflexes, motor functions, anxiety, spatial learning and short-term memory as the wild-type mice [84]. No significant role in neurogenesis or neuron proliferation has been observed in another study in which only 8.5-month-old males were studied [85]. All these data suggest that ABCA7 might play a minor role in behavioral domains, but again further studies considering mouse strains, ages, sex or methodology are necessary to elucidate the role of ABCA7 in brain development and behaviors.

In humans, it was demonstrated more recently in brain samples that levels of ABCA7 modestly but significantly decrease with normal aging [23]. A significant association between ABCA7 SNP (rs3764650) and cognitive decline was only observed in females in a longitudinal study including 3267 females and 3026 males [70].

### 4.3. Impact of ABCA7 Depletion in Aβ Burden in Animals and Cells

When KO animals are crossbred with transgenic mice overproducing Aβ peptides (J20 mice at ± 17 months of age), Aβ burden was worsened, reinforcing the link between ABCA7 and AD. Interestingly, this effect is rather the consequence of a higher Aβ peptide accumulation than an overproduction [86]. Indeed, a significant decrease of Aβ peptide efflux across the BBB was reported in an *Abca7*-deficient in vitro model of the BBB, in relation to ApoA-I lipidation status [20], as well as a decrease in the phagocytosis process mediated by microglial cells [81,86]. These cells express high levels of ABCA7 when compared with neurons [82]. In addition, it was reported that *Abca7* haplodeficiency provokes a microglial abnormal morphology and an altered response to inflammation, thus leading to cerebral amyloid accumulation in mice [87].

On the contrary to the aforementioned study demonstrating that *Abca7* deficiency does not impact Aβ peptide synthesis [86], other studies reported that the absence of *Abca7* in AD transgenic mouse models (APP/PS1 and TgCRND8) promotes the Aβ peptide production [22,83] or that ABCA7 upregulation in vitro diminishes this synthesis [82]. Transgenic models used to reproduce AD in mice should be taken into account and probably explain why such discrepancies are observed. It is important to note that in AD mouse models, ABCA1 and ABCA7 expressions seem to act in the same direction in order to modulate Aβ peptide production and deposition.

### 4.4. Evidence in AD Patients

Numerous works have studied ABCA7 as a risk gene in patients with mild cognitive impairment (MCI), or in AD individuals. All these studies are summarized elsewhere [57], and Table 1 gives an overview of AD-associated ABCA7 (epi-) genetic variation. Recently, an analysis of cerebrospinal fluid (CSF) from AD patients bearing the protective rs3764650 allele reported a decrease of CSF Aβ_1-42_ although total tau and phosphorylated tau levels remained unchanged [63]. Another recent study investigating genetic associations correlating with Aβ deposition found a strong association with ABCA7 among 1600 investigated genes [88]. These observations confirmed the link between ABCA7 and Aβ peptides in humans but do not explain if this is mainly due to a decrease in Aβ production, or an increase of the clearance mediated by microglia across the BBB. A recent and elegant study from Lyssenko et al. investigated ABCA7 protein level in different Braak stages in AD patients and controls [23]. They observed that patients with low levels of ABCA7 are more prone to develop early AD than patients with the highest ABCA7 levels. Therefore, authors suggested that ABCA7 acts like a blocker of AD in the early stage of disease, particularly by removing toxic lipids from cellular membranes. As largely demonstrated now, lipid composition of cellular membranes can influence secretase activities and then APP processing and Aβ synthesis [89], therefore it might be hypothesized that ABCA7 levels and functioning can directly modulate APP processing and then, Aβ production. Another recent study performed in the frame of the Alzheimer’s Disease Neuroimaging Initiative (ADNI) suggests also that ABCA7 acts very early in amyloid deposition [76]. Authors analyzed 18F-florbetapir positron emission tomographic data from 322 cognitively normal control individuals, 496 patients with mild cognitive impairment (MCI), and 159 individuals with AD. These results were compared with genetic data obtained in GWAS. They clearly observed that several AD risk variants of ABCA7 are highly associated with increased amyloid deposition, in particular in the cognitively healthy and MCI populations, but not in AD patients [76].

However, the impact of these variants on ABCA7 expression is still unclear since some studies report that deleterious alleles such as rs4147929 downregulates ABCA7 expression in AD patients [57], whereas other studies report contradictory results [58]. Studies investigating the association between rs3764650 and ABCA7 expression also reported inconsistent findings [65,90]. Therefore, there is a need of further studies that should address how ABCA7 is regulated and how this transporter is involved in brain functioning as well in Aβ peptide clearance or deposition in AD brains. Use of iPSCs cells from patients bearing SNPs of ABCA7 should open new avenues for investigating this transporter in AD. These cells can be differentiated into neurons to investigate not only neurogenesis but also Aβ production. It would be also possible to generate in vitro models of the BBB [91] or microglial cells [92] to further study Aβ clearance from CNS, and therefore to gain insight into the molecular role of ABCA7 in AD.

## 5. ABCA7 and Cancers

As mentioned previously, due to the GWAS studies, ABCA7 functions were mainly studied in AD mouse models and in AD samples. This is noteworthy because ABC transporters have been closely linked to the MDR phenotype observed in cancer cells in the most recent studies of ABCA7 in cancer. Finally, no relation with the MDR phenotype has been observed, but a possible involvement in the epithelial to mesenchymal transition (EMT process) was reported, in particular in ovarian cancers (OC) [93]. In EMT, cells lose their epithelial markers and express more mesenchymal markers in order to acquire capacities like migration, invasion and proliferation, characteristics of malignancies and metastatic cancers. In this study, authors demonstrated that ABCA7 was upregulated in ovarian cancer (OC) cells from patients when compared to adjacent non-cancer tissues [93]. This upregulation was associated with poor survival rates in OC patients. When ABCA7 expression is suppressed, OC cell lines showed a decrease in migration and an increase in epithelial marker expression such as E-cadherin, correlated with a decrease of the mesenchymal marker, N-cadherin [93,94]. Interestingly, ABCA7 knockdown decreased the TGF beta transcription factor SMAD-4, a key regulator for EMT [93].

Additionally, regulation of ABCA7 in cancer cells was reported to be modulated by the micro-RNA tumor suppressor called Mir-197-3p. This is found downregulated in OVACAR-3 cells as well as in other types of cancer cells such as hepatocarcinoma cells [94], suggesting a potential implication of ABCA7 in other types of cancers. Further investigations for the ABCA7 role in cancer are needed for a better understanding of this transporter functions and for a better diagnosis and prognosis. They will also determine if targeting ABCA7 expression might be a promising approach to prevent or cure certain types of cancers.

## 6. Conclusions and Future Perspectives

**Figure 3:**
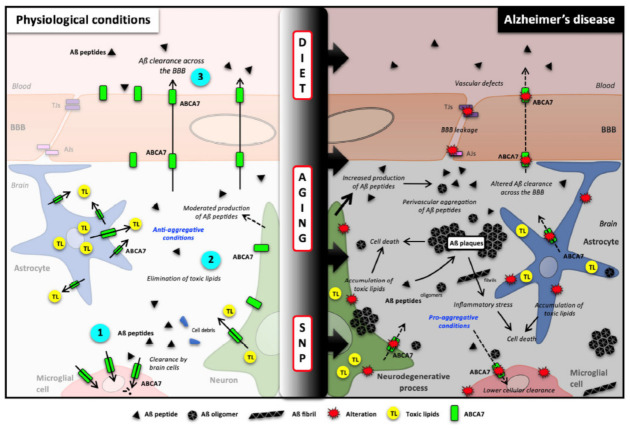
Potential roles of ABCA7 in brain cholesterol homeostasis and Aβ clearance and synthesis. (**Left**), in healthy brains, ABCA7 is normally expressed by neurons, glial cells and blood–brain barrier endothelial cells where it mediates Aβ clearance (1 and 3) as well cellular and toxic lipid (TL) efflux (2). Because Aβ peptide production is also linked with cellular lipid levels, it is likely that ABCA7 can also regulate Aβ synthesis. (**Right**) Single nucleotide polymorphisms (SNPs), aging and diet can affect ABCA7 function and expression, thus downregulating Aβ peptide clearance, decreasing toxic lipid recycling and then promoting Aβ burden. Aβ peptides: amyloid-β peptides; ABCA7: ATP-binding cassette subfamily A member 7; AJs: Adherent junctions of the BBB; BBB: blood–brain barrier; SNP: single nucleotide polymorphism; TJs: tight junctions of the BBB; TL: toxic lipids.

As summarized in Figure 3, recent evidence supports a central role of ABCA7 in AD. Increase of ABCA7 expression in the first step of the disease would help to maintain the cerebral lipid homeostasis and the balance between the amyloid synthesis and clearance. This latter process is controlled by microglial cell degradation and elimination across the blood–brain barrier. Aging, genetic polymorphisms and probably external factors such as diet or physical activity, influence ABCA7 expression, thereby promoting or slowing down amyloid deposition and then AD onset and evolution (Figure 3).

To exploit ABCA7 for therapeutic purposes, more attention needs to be given to its role in phagocytosis processes, in particular at the level of microglial cells that remove amyloid and play a central role in inflammatory processes. Then, it might be essential to better characterize ABCA7 regulatory sequences and signaling pathways in order to develop new molecules or approaches able to promote its cerebral expression, in healthy and AD patients. It might be also suggested that development of new tracers for measuring ABCA7 activity at the brain level should be useful to detect AD or other neurodegenerative diseases at early stages.

At last, gender differences reported in several studies might also be taken into account to understand if ABCA7 has a different role in females rather in males, as highlighted by the role of ABCA7 in ovarian cancer [93]. This hypothesis is also strongly supported by the fact that AD deficiencies in mouse models affect more females than males [95,96] and that cognitive decline has been observed in females bearing ABCA7 SNPs when compared with males [70].

## Figures and Tables

**Table 1:** AD-associated ABCA7 (epi-)genetic variation and their effects in individuals.

Variation	Interpretation	Reported Significant Effect of the Risk Allele	
Common Risk-Increasing Variants		Amyloid and Tau pathology	Brain Morphology and Clinical Symptoms
rs3764650	Intronic GWAS SNP, low predicted functional effect	- Increased neuritic plaque burden [62] - Decreased CSF Aβ_1-42_ levels [63].	- Cortical and hippocampal atrophy [64] - Later age at onset and shorter disease duration [65] - Interaction on memory [66] - Association with posterior cortical atrophy variant of AD [67,68] - Memory decline for subjects who eventually developed MCI/LOAD [69] - Cognitive declines in females [70] - Lower immediate recall test and reduced rate of decline in symbol digit modalities test [71]
rs4147929	Intronic GWAS SNP, low predicted functional effect		- Voxel-based morphometry in the left postcentral gyrus [72] - Brain asymmetry in the hippocampus [73] - Increase of symptomatic AD compared to asymptomatic [74]
rs3752246	Missense GWAS SNP, predicted benign	- Increased amyloid deposition [75] - Increased brain amyloidosis [76]	- Decreased mean medial temporal lobe gray-patter density in dementia patients [77] - Interaction on memory [66]
rs115550680	Intronic GWAS SNP, low predicted functional effect		- Dissociation in entorhinal cortex resting state functional connectivity [78] - Behavioral generalization [78]

rs78117248	Intronic GWAS SNP, low predicted functional effect		
rs142076058	Loss-of-function		
ABCA7 VNTR expansions	Reduced ABCA7 expression, loss of exon 19 encoding an ATP-binding domain		
**Common Protective Variants**		**Amyloid and Tau pathology**	**Brain Morphology and Clinical Symptoms**
rs72973581	Missense variant		
**CpG Methylation**		**Amyloid and Tau pathology**	**Brain Morphology and Clinical Symptoms**
cg02308560	Hypermethylation in AD, effect on ABCA7 unknown		
cg24402332			
cg04587220		- Increased brain amyloidosis and higher tau tangle density [79]	
**Rare Variants**		**Amyloid and Tau pathology**	**Brain Morphology and Clinical Symptoms**
Missense and PTC variants	Loss-of-function for PTC variants. Unclear for missense variants.		

Variants and CpG methylation sites in ABCA7 with their interpreted functional effect, and their association with AD and brain morphology and clinical symptoms when investigated. Adapted from [57]. AD: Alzheimer’s disease; GWAS: Genome-wide association studies; PTC: premature termination codon; SNP: single nucleotide polymorphism; VNTR: variable number tandem repeat.
